# Risk factors for wound-related complications after surgical stabilization of spinal metastases with a special focus on the effect of postoperative radiation therapy

**DOI:** 10.1186/s12893-021-01431-9

**Published:** 2021-12-17

**Authors:** Jan-Sven Jarvers, Maximilian Lange, Samuel Schiemann, Jan Pfränger, Christoph-Eckhard Heyde, Georg Osterhoff

**Affiliations:** Department of Orthopaedics, Trauma and Plastic Surgery, University Hospital Leipzig, 04103 Leipzig, Germany

**Keywords:** Spine, Metastastatic bone disease, Surgery, Radiotherapy, Wound infection, Surgical site infection, Risk factors, Wound complications

## Abstract

**Background:**

Advancements in the field of oncological therapies during the last decades have led to a significantly prolonged survival of cancer patients. This has led to an increase in the incidence of spinal metastases. The purpose of this study was to assess risk factors for wound-related complications after surgical stabilization of spinal metastases with a special focus on the effect of postoperative RT and its timing.

**Methods:**

Patients who had been treated for metastatic spine disease by surgical stabilization followed by radiotherapy between 01/2012 and 03/2019 were included and a retrospective chart review was performed.

**Results:**

Of 604 patients who underwent stabilizing surgery for spinal metastases, 237 patients (mean age 66 years, SD 11) with a mean follow-up of 11 months (SD 7) were eligible for further analysis. Forty-one patients (17.3%) had wound-related complications, 32 of them before and 9 after beginning of the RT. Revision surgery was necessary in 26 patients (11.0%). Body weight (p = 0.021), obesity (p = 0.018), ASA > 2 (p = 0.001), and start of radiation therapy within 21 days after surgery (p = 0.047) were associated with an increased risk for wound complications. Patients with chemotherapy within 3 weeks of surgery (12%) were more likely to have a wound-related surgical revision (p = 0.031).

**Conclusion:**

Body weight, obesity and ASA > 2 were associated with an increased risk for wound complications. Patients with chemotherapy within 3 weeks of the surgery were more likely to have a wound-related revision surgery. Patients who had begun radiation therapy within 21 days after surgery were more likely to have a wound complication compared to patients who waited longer.

## Background

The progress and innovations in the field of oncological therapies during the last decades have led to a significantly prolonged survival of cancer patients. At the same time, this development is associated with an increased prevalence of metastatic disease among the world’s population. About 70% of the secondary malignant skeletal manifestations are located in the spine [1]. Metastatic spinal cord compression as a complication of metastatic spine disease (MSD) occurs in at least 10% of these patients [1, 2]. Main indications for a surgical intervention are pathological fractures, severe pain, and neurological deficits caused by spinal cord compression [3].

For most tumor entities, the common treatment of symptomatic MSD consists of surgery in combination with radiotherapy (RT) and chemotherapy. The goal of combining surgery and RT is to avoid local recurrence of MSD and to improve the patient’s quality of life in the long term [3]. Even in the setting neurological deficits due to MSD, a combined approach showed better results compared to RT alone [4]. In addition, better patient survival and cost savings were demonstrated throughout the treatment period [5, 6].

The earlier RT is applied, the better local tumor control will be. One of the downsides of early RT is the increased likelihood of postoperative wound-related complications [4, 7]. In particular, the proliferation phase of wound healing can be affected by the damaging effect of ionizing radiation on fibroblasts and their growth [8]. Likewise, the risk of infection for wounds in additionally irradiated skin areas is increased, since the inflammatory and local leukocyte reaction proceeds at a slower rate after RT. As in people with peripheral arterial disease or diabetes, tissue hypoxia is observed with RT, resulting in a decreased ability to fight bacterial contamination [9].

In consequence, patients with a short time interval between surgery and RT are likely to have a higher rate of wound complications [10, 11]. In addition, a variety of other risk factors such as obesity and diabetes act as predisposing factors [12].

Hence, the purpose of this study was to assess risk factors for wound-related complications after surgical stabilization of spinal metastases with a special focus on the effect of postoperative RT and its timing.

## Methods

A monocentric retrospective cohort study was conducted at a university level 1 spine center through review of charts. This study was approved by the local institutional ethics committee (reference 027/21-ek).

### Patients

**Fig. 1:**
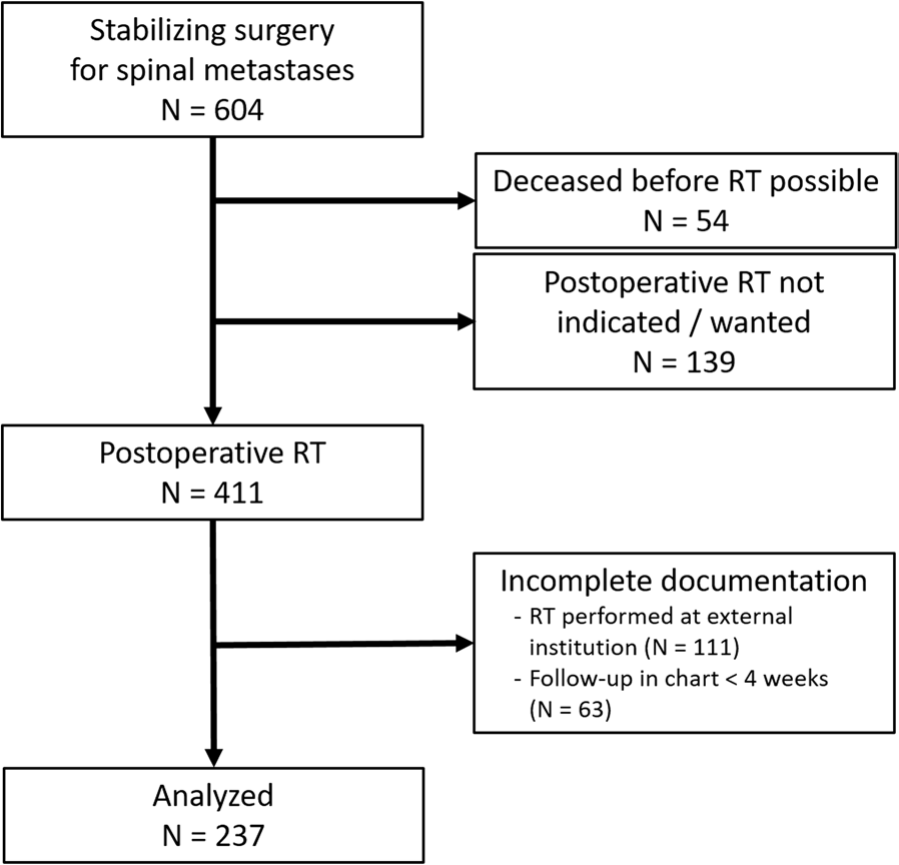
Patient inclusion flow chart. *RT* radiation therapy

All consecutive patients aged 18 or older who were treated for metastatic spine disease by surgical stabilization between January 2012 and March 2019 were identified (N = 604). Patients who received no radiation therapy (RT) or had incomplete documentation or a follow-up of less than 4 weeks after surgery were excluded leaving 237 patients for final analysis (Fig. 1). Indications for surgical intervention were pathological fractures leading to instability, neurological deficits due to metastatic spinal cord compression, and persistent tumor pain. As a standard, all patients received an intravenous antibiotic prophylaxis with cefuroxime or—in case of known allergy—clindamycin at 30 min before incision. For patients with spinal metastases from level T1 to S1 (N = 219), the standard open intervention was performed in prone position by a posterior approach to the spine with fixation of the affected segments by internal pedicle screw/rod fixation and optional additional laminectomy or hemilaminectomy for spinal cord or nerve root decompression. A posterior approach was also commonly chosen for metastases of the cervical spine (N = 14). However, cervical vertebral body resection via an anterior approach was performed in four cases. Wound drains were used in all cases and left in situ for 24–72 h.

All patients included received individualized conventional radiotherapy according to established treatment regimens by the institutional university department of radiation oncology.

### Data acquisition

Patient-related variables on epidemiology, pre-exiting conditions including the American Society of Anesthesiologists’ risk classification (ASA), medication, type of primary tumor, localization of the metastases, and data regarding the surgical and oncological procedures were obtained by a retrospective chart review. In addition, the occurrence of wound complications and the need for surgical revisions within 6 months after surgery were documented. Wound complications were defined as either surgical site infections or any delay in healing of the wound > 3 weeks. In accordance with the CDC criteria, surgical site infections were defined as wounds that showed one of the following criteria: (1) purulent drainage, or (2) spontaneous dehiscence or surgical revision and detection of microorganisms in the microbiological culture and fever or local tenderness, or (3) an abscess or other evidence of infection involving the incision that is detected on gross anatomical or histopathologic exam, or imaging test [13].

### Statistical analysis

All data were recorded in an Excel database (Microsoft Corp., Washington, DC, USA) and exported to SPSS 26.0 (SPSS Inc., Chicago, IL, USA) for statistical analysis. Unless otherwise denoted, data was summarized as mean with standard deviation (SD) or frequencies and percentages (%).

Primary outcome was the occurrence of wound-related complication within 6 months after surgery. Where applicable, nominal variables crosstabs were associated using Chi-Square or Fisher’s Exact tests. Nonparametric tests were used to compare continuous data. The level of significance was defined as *p* < 0.05.

## Results

In total, 237 patients (84 female, mean age 66 years, SD 11) were available for final analysis. The mean follow-up was 11 months (SD 7). Forty-one patients (17.3%) had wound-related complications that only required prolonged hospitalization in 15 cases (6.3%) and an additional revision surgery in 26 patients (11.0%). Revision surgery was performed mean 27 days (SD 24, range 7–112) after the initial surgery. In 21 patients (8.9%), a pathogenic microorganism was found in the wound with *Staph. epididermidis* (n = 8) and *Staphylococcus aureus* (n = 8) being the most frequent ones.

### Patient-related risk factors

Body weight (p = 0.021), obesity (BMI > 30 kg/m2, p = 0.018), ASA > 2 (p = 0.001), and anticoagulant medication (p = 0.045) were associated with an increased risk for wound complications (Table 1). In addition, patients who had received chemotherapy within 3 weeks of the surgery (29/237, 12%) were more likely to have a wound-related revision surgery compared to patients who were not under chemotherapy (22% vs. 9%, p = 0.031).

**Table 1:** Patient-related risk factors

	Wound complication		*p*	Total
	No	Yes		
N	196	41		237
Age [y (SD)]	66 (12)	67 (10)	0.703^†^	66 (11)
Female sex	73 (37%)	11 (27%)	0.138*	84 (35%)
Height	171 (9)	173 (8)	0.377	172 (9)
Body weight	77 (13)	82 (16)	0.021^†^	78 (14)
Obesity [BMI > 30 kg/m^2^]	31 (16%)	18 (44%)	0.018*	49 (21%)
ASA > 2	87 (44%)	30 (73%)	0.001*	117 (49%)
Comorbidity				
Diabetes	41 (21%)	14 (34%)	0.056*	55 (23%)
Chemotherpy^a^	27 (14%)	10 (24%)	0.076*	37 (16%)
Smoking	26 (13%)	6 (15%)	0.491*	32 (14%)
Anticoagulant medication	51 (26%)	7 (17%)	0.045*	58 (24%)

*ASA* American Society of Anesthesiologists’ risk classification, *BMI* body mass index

^†^Mann–Whitney-U-Test. *Pearson Chi-square/Fisher’s exact test

^a^Within 3 weeks before/after surgery

### Surgery-related risk factors

The mean duration of the surgical intervention was 168 min (SD 82) from incision to suture with mean 5.4 (SD 2.2) vertebral bodies being instrumented. Open surgery was performed in 205 cases (86%), percutaneous instrumentation was done in 30 cases (13%), and percutaneous KP/VP in 2 patients (1%). Decompression by (hemi-)laminectomy was performed in 184 cases (78%). There was no significant association between the occurrence of any wound complication and surgical duration (p = 0.126), the number of instrumented vertebral bodies (p = 0.642), or whether the intervention was performed through an open or percutaneous approach (p = 0.054).

However, patients who had undergone a (hemi-)laminectomy were more likely to require revision surgery than patients without such an additional procedure (15% vs. 4%, p = 0.044). Three patients (1%) suffered an incidential durotomy during the initial intervention, requiring revision surgery in one patient.

### Radiation-related risk factors

All patients received postoperative radiation therapy mean 39 days (SD 29) after surgery. The mean single dose applied was 2.8 Gy (SD 0.6) resulting in a total dose of 31.4 Gy (SD 6.7).

Of the 41 wound complications observed in this cohort, only 9 (22%) were observed mean 38 days (SD 21, range 5–78) after beginning of the radiation therapy. Hence, patients with a wound complication prior to radiation therapy were excluded from further analysis. Among the remaining 205 patients, those with a wound complication had a significantly shorter waiting time between surgery and radiation compared to those without a complication (23 days, SD 7 vs. 40 days, SD 30, p = 018). Patients who had begun radiation therapy within 21 days after surgery were more likely to have wound complication compared to patients who had waited longer (p = 0.047).

## Discussion

Spine surgery for metastatic bone lesions is associated with a significant risk for postoperative complications regarding the surgical wound site resulting in longer hospital stays, unplanned reoperations, poor neurological outcomes and significant morbidity [14–16].

The purpose of this study was to assess risk factors for wound-related complications after surgical stabilization of spinal metastases with a special focus on the effect of postoperative radiation and its timing.

In the present study, body weight, obesity, and ASA were associated with an increased risk for wound complications. Patients who had received chemotherapy within 3 weeks of the surgery were more likely to have a wound-related revision surgery. These findings are in line with data summarized in a recent systematic review on wound-related complications after surgery for metastatic spine lesions by Schilling et al. [17]. In addition, complication rates after spine surgery have been associated with female sex, smoking history, preoperative radiotherapy, corticosteroid use, previous spine operations, transfusion rates, postoperative delirium, dysphagia and incidental durotomy [18–21].

While patients with (hemi-)laminectomy were more likely to require revision surgery in the present study, other surgery-related risk factors like duration of surgery, number of instrumented levels, or open versus percutaneous approach were not found to have a significant effect on the occurrence of wound-related complications. This is in contrast to the current literature. In a comparative study of open versus MIS by Kumar et al. [22] it was shown that the postoperative infection rate was 3% in the MIS group versus 16% in the open surgery group. It was also noted that patients undergoing MIS had earlier wound healing, hence, allowing earlier introduction of RT for residual local disease control [23]. However, Kumar et al. included all kind of patients with metastatic spine tumors while the present study focused on patients who received surgery and RT.

Beyond the parameters investigated in this study, it has been discussed that pre-existing venous thromboembolisms (VTE) increase the risk for wound complications [24–26]. This could be explained by the fact that patients developing VTE are treated with anticoagulants, thereby delaying wound healing. It is in line with the significant correlation between wound complications and anticoagulation that was found in the present study.

In our patient cohort, patients with a wound complication had a significantly shorter time interval between surgery and radiation compared to those without a complication. Patients who had begun radiation therapy within 21 days after surgery were more likely to have wound complications compared to patients who had waited longer. A cutoff of 21 days was chosen as it resembles the clinical routine at the authors’ institution with suture removal in the outpatient clinic 2 weeks after surgery and start of radiation a week later. It has been well described that both, pre- and post-operative RT can affect surgical wound healing [7, 27]. Ionising radiation impairs fibroblast function and has a negative impact on their growth [8]. Such fibroblast depletion may account for the effects of radiation such as dermal atrophy, wound contraction, and a predisposition to necrosis [28]. Radiotherapy also increases the frailty of wounds to infection by suppressing the inflammatory response, hindering antibody production, and reducing the immediate leukocyte response [9]. Irradiated skin wounds are hypoxic and are therefore unable to counteract bacterial contamination.

The current literature clearly underlines the fact that postoperative RT is associated with a lower rate of wound infections compared to preoperative RT. Laohacharoensombat et al. [29] only saw wound infection in one out of thirty patients who received RT 14 days after surgery. Berriochoa et al. [30] also reported a low incidence of wound complications in their retrospective study of patients undergoing RT within 3 months after surgery. Nevertheless, several studies favor preoperative radiation.

Various authors investigated what duration between pre-operative RT and surgery, which ranges from 1 day to 6 weeks. Ghogawala et al. [25] reported a wound complication rate of 46% when the surgery was performed ≤ 7 days after RT, as compared to 20% when surgery was performed > 7 days after RT. Lee et al. [31] concluded that surgery should be performed 2 weeks after RT, with a minimum interval of 1 week. This topic still is discussed controversially, as there are several studies figured out preoperative radiation as a factor for wound complications [24, 32–34]. The optimal time between surgery and post-operative RT, however, remains debated. In our therapeutic regime we only use postoperative RT.

Of note, most postoperative wound complications (78%) occurred before RT had begun. This underlines the fact that wound complications in patients with metastatic bone disease are the result of multiple factors and that RT is only one of them.

This study offers some limitations associated with its retrospective design. The relatively short follow-up period of one year is a result of the patients’ advanced general condition in context of the tumor disease. However, with wound-complications being the primary outcome a follow-up of one year seems sufficient. No distinction between different tumor entities and their grading was made and it is possible that are more aggressive tumors may affect wound-healing directly—or vice versa as RT can be more effective in more aggressive entities.

The findings of this study may allow patients and surgeons to address modifiable risks when preparing for operations and postoperative radiation therapy. Future prospective comparative studies need to further investigate the risk factors for wound complications and the ideal duration between surgery and radiation therapy. It is very likely that different time thresholds may apply for different individual patterns of risk factors. Further evidence about the risks of wound complications can influence common clinical decisions such as the cost benefit analyses of adjuvant therapies or the decision to pursue palliative operations.

## Conclusion

Body weight, obesity and ASA > 2 were associated with an increased risk for wound complications. In addition, patients who had received chemotherapy within 3 weeks of the surgery were more likely to have a wound-related revision surgery compared to patients who were not under chemotherapy. It was found that patients who had begun radiation therapy within 21 days after surgery were more likely to have wound complications compared to patients who waited longer.

## Data Availability

Anonymized grouped data available upon request from the corresponding author.

## Abbreviations

ASA: American Society of Anesthesiologists’ risk classification
BMI: Body mass index
MIS: Minimal-invasive surgery
RT: Radiation therapy
SD: Standard deviation
VTE: Venous thromboembolisms
